# “One Size Fits All” Doesn’t Fit When It Comes to Long-Term Opioid Use for People with Chronic Pain

**DOI:** 10.1080/24740527.2017.1319733

**Published:** 2017-05-04

**Authors:** M. E. Lynch, J. Katz

**Affiliations:** a Departments of Anesthesiology, Pain Medicine & Perioperative Care, Psychiatry, and Pharmacology, Dalhousie University, Halifax, NS, Canada; b Department of Psychology, York University, Toronto, ON, Canada

The use of opioids in chronic non-cancer pain continues to be a flashpoint in medicine. The media and others continue to cite rising deaths due to opioids without clarifying that a large percentage of the current deaths are related to illicit highly potent fentanyl and other synthetic opioids coming across our borders from China. This misunderstanding is leading to an increasingly harsh regulatory climate for physicians and many physicians are electing to avoid opioid prescribing completely. Further, in this environment of fear, there are many instances of physicians refusing to treat people with chronic pain. This is unethical and unacceptable but one can begin to understand given the inappropriate reactions of regulators across the country.

In British Columbia the College of Physicians and Surgeons released professional standards and guidelines in June 2016 dealing with the prescribing of drugs with potential for misuse and diversion. The standard supported the controversial maximum dose guidelines published by the US Center for Disease Control (CDC) by stating that doses greater than 50 morphine milligram equivalents (MME) per day warrant careful reassessment and documentation and that doses greater than 90 MME per day warrant substantive evidence of exceptional need and benefit as well as advising against long term opioids in individuals with certain types of pain including headache, fibromyalgia, and axial low back pain. The guideline section of this document also recommended against use in people with mental health issues or psychiatric disorder and young people. One must use care in these populations but to recommend against opioids for whole diagnostic categories is not evidence-based and is discriminatory. It is also non-pharmacological to recommend the same dose for all people regardless of body mass or age.

Last year several other Canadian provinces also adopted the CDC guidelines rather than wait for the update on the 2010 Canadian National Opioid guidelines^2^ scheduled for release early this year. This includes Nova Scotia where there are an increasing number of “Explain” letters and phone calls from our provincial Prescription Monitoring Program going out to physicians requesting explanation/justification of their use of opioids in patients on doses above the maximum recommended by the CDC. Some of this correspondence has been directed to me (M.L.) regarding my patients. Two examples are women with the most severe rheumatoid arthritis I have seen in my 25-year career treating patients with chronic pain, both of whom would not have been able to mobilize at all without the assistance of the opioid.

In Ontario, the College of Physicians and Surgeons sent out 86 simultaneous “Section 75 investigation” notifications to physicians who have at least 8 patients on a dose above 850 mg oral morphine equivalents of opioid therapy per day.^3^ This involves a compulsory 20–25 patient chart review that takes a long time to complete and is labor-intensive for busy clinicians. It has been called an “educational” exercise but the physicians find it very intimidating. Add to this the Opioid Summit co-hosted by the Federal Minister of Health, which took place November 18–19, 2016 in Ottawa, Ontario. At this summit there was no one on the agenda to present from the person-with-pain perspective. In addition, there was not one medical professional specialized in the management of pain on the speakers list. Fortunately, there were 2 people with pain in the audience and members of the Canadian Pain Society (CPS) managed to get the incoming president of CPS on the invitation list days before the conference. The agenda was focused on opioid-related harms and addiction so the voice of those with pain was at a significant disadvantage.

This brings us to the most recent Canadian draft recommendations for the use of opioids in chronic non-cancer pain.^4^ Our understanding from direct communication with the Chair of the committee is that individuals on the committee were excluded from voting on recommendations if they had either a financial or intellectual conflict of interest. Avoidance of a financial conflict of interest is necessary, but it appears that the principle of avoidance of an intellectual conflict of interest led to a significant imbalance of voting members with any clinical experience or training in the management of chronic pain. This is inappropriate as these guidelines will have a significant impact on people with pain as well as the clinicians assisting them who, we argue, should have been more involved in the creation of the guidelines. Experienced pain clinicians were represented on a “Clinical Expert Committee” but they did not have a vote and their role was limited to drafting clinical guidance material regarding how physicians can put the guidelines into practice. In our opinion, pain clinicians should have been involved as voting members on the guidelines, in this way the guidelines would have been informed by clinical experience as well as the evidence in the literature. This balance is critical in the practice of good medicine.

Table 1 lists the 10 draft recommendations as well as our proposed modifications to the recommendations. As they stand now the draft recommendations for the use of opioids in chronic non-cancer pain are regressive. The previous National Guideline^2^ was far superior with helpful guidance to clinicians as well as providing additional resources. The new draft recommendations have stated the obvious (e.g., use nonpharmacological or nonopioid treatments first), made recommendations that are discriminatory and often not possible to implement (e.g., stabilize the co-morbid psychiatric disorder first before starting an opioid), and decreased the maximum daily dose of opioid to less than a significant subgroup of people will require (less than 50 mg morphine equivalents per day). Our concern is that if these recommendations are accepted the collateral damage to people with pain will escalate. We are hopeful that there will be significant input from the people with pain and experienced clinicians so that the final guidelines will be significantly improved.

**Table 1.** Present authors’ proposed modifications to the 2017 draft recommendations for use of opioids in chronic non-cancer pain.^4^

Number and Recommendation	Proposed Modification to Draft Recommendation
1 – Strong recommendation	No modification
2 – Weak recommendation	No modification
3- Strong recommendation (Against)	For patients with co-morbid serious chronic pain and substance use disorder, an opioid may still be considered but all efforts should be made to consult an addictions specialist and put in place appropriate structure (eg. supervised daily dispensing) to assure patient safety.
4 – Weak recommendation	For patients with co-morbid serious psychiatric disorder and chronic non-cancer pain, where it is not possible to stabilize the psychiatric disorder first, an opioid trial may be considered as long as the psychiatric condition is also being addressed.
5 – Weak recommendation	For patients with co-morbid serious chronic pain and a distant past history of a substance use disorder, an opioid may still be considered. The physician must assess the patient’s current level of safety with opioids and put into place appropriate structure to assure there will be no risk of relapse. The need for involvement with an addictions specialist should be discussed.
6 – Weak recommendation	The lowest effective dose should be used.
7 – Strong recommendation	For patients who have obtained a partial benefit and where there are no limiting side effects, a trial of higher dose therapy may be considered. In this case patients should be monitored more closely (e.g., weekly). For patients requiring more than 90 mg oral morphine equivalent per day referral to a pain specialist should be considered.
8 – Weak recommendation	No modification
9 – Weak recommendation	If opioid rotation is not helpful in improving pain control or reducing problematic side effects and if a trial of higher dose opioid therapy leads to no further improvement in pain or a worsening of side effects, the dose of opioid should be reduced to the dose that was associated with maximal benefit and minimal side effects. If there is no appreciable benefit this is a failed trial and the patient should taper and discontinue the opioid.
10 – Weak recommendation	If patients have experienced a reduction in pain without problematic side effects on a stable dose of opioid, and if a reduction in the dose leads to increased pain and a poorer quality of life then the dose of opioid should be increased back to the dose where pain was most effectively controlled.

In examining this issue several additional key points that are important to consider.

## A significant subpopulation of people with chronic pain benefit from opioids with a reduction in pain and their access should be preserved

The National Neuropathic Pain Database group identified that 17.9% of patients with neuropathic pain experienced more than a 30% reduction in pain with a concomitant reduction in pain-related interference, taking a mean daily dose of 81.7 mg oral morphine equivalents.^5^ Watson et al. found opioids to be safe and effective in a survey of 84 selected patients with intractable non-cancer pain taking a median dose of 220 mg oral morphine equivalents per day for the whole group and 510 mg per day for the largest group of people with back and neuropathic leg pain.^6^ A Cochrane database review of long-term opioid management for chronic non-cancer pain identified 26 studies involving 4,893 patients and examined 3 routes of delivery (per os, transdermal, and intrathecal).^7^ All studies demonstrated efficacy and addiction indicators were present in only 0.27%. If we assume that there were in fact no cases of addiction (see Table 2 for definitions) among the studies that did not report whether addiction occurred (as it seems likely that such an important adverse event would be reported if it were observed), then the rate is 0.14% . The authors concluded that strong opioids in well-selected patients with no addiction or substance abuse history can lead to long-term pain relief for some patients with a very small risk of developing addiction, abuse or other serious side effects. The authors also cautioned that these rates should not be generalized to an unselected population or to individuals taking opioids without appropriate medical supervision.^7^

**Table 2.** Definitions of substance use terms.

Term	Definition	Source
Addiction	A primary, chronic, neurobiological disease with genetic, psychosocial and environmental factors influencing its development and manifestations. It is characterized by one or more of impaired control, compulsive use, continued use despite harm and craving	Liaison Committee on Pain and Addiction^8^
Opioid Use Disorder	A minimum of 2–3 criteria is required for a mild substance use disorder diagnosis, while 4–5 is moderate, and 6– is severe. Opioid Use Disorder is specified instead of Substance Use Disorder, if opioids are the drug of abuse. Note: A printable checklist version is linked below: Taking the opioid in larger amounts and for longer than intended Wanting to cut down or quit but not being able to do it Spending a lot of time obtaining the opioid Craving or a strong desire to use opioids Repeatedly unable to carry out major obligations at work, school, or home due to opioid use Continued use despite persistent or recurring social or interpersonal problems caused or made worse by opioid use Stopping or reducing important social, occupational, or recreational activities due to opioid use Recurrent use of opioids in physically hazardous situations Consistent use of opioids despite acknowledgment of persistent or recurrent physical or psychological difficulties from using opioids *Tolerance as defined by either a need for markedly increased amounts to achieve intoxication or desired effect or markedly diminished effect with continued use of the same amount. (Does not apply for diminished effect when used appropriately under medical supervision) *Withdrawal manifesting as either characteristic syndrome or the substance is used to avoid withdrawal (Does not apply when used appropriately under medical supervision) *This criterion is not considered to be met for those individuals taking opioids solely under appropriate medical supervision.	DSM-5^9^
Opioid misuse NSDUH	Use in any way not directed by a doctor, including … use in greater amounts, more often, or longer than told to take a drug	*NSDUH^18^
Opioid use to get high CTADS	Use of an opioid in order to get high	**CTADS^19^
Physical dependence	A state of adaptation manifested by a drug class-specific withdrawal syndrome that can be produced by abrupt cessation, rapid dose reduction, decreasing blood level of the drug and/or administration of an antagonist	Liaison Committee on Pain and Addiction^8^
Tolerance	A state of adaptation in which exposure to the drug results in changes that result in diminution of one or more of the drugs effects over time	Liaison Committee on Pain and Addiction^8^
Opioid use	Use of an opioid as opposed to a disorder (see definition above)	

*NSDUH = US National Survey on Drug Use and Health. **CTADS = Canadian tobacco, alcohol and drug use monitoring survey.

A qualitative study examining the lived experience of adults using prescription opioids to manage chronic pain found benefits outweighed the negative effects and that most of the negative effects were socio-culturally induced including participants describing guilt and stigmatization for using opioids.^10^ Patients in this study commented:

“I have to say these drugs give me a quality of life that I did not think I would ever have again, it allows me to live my life to the fullest that I can” (p.17).

“To tell you the truth, I probably wouldn’t be here right now if I was not taking it. I probably wouldn’t be able to walk or get out of bed” (p. 17).

“There would be no life if I didn’t have this and I thank God for them because without them I’d be… well… I wouldn’t be. I just couldn’t go on. I would have committed suicide long ago” (p. 20).

## Appropriate and responsible medical exposure to opioids for chronic pain does not lead to addiction

Recent evidence has demonstrated a very low risk of persistent opioid use after medical exposure to opioids following major elective surgery. Of 39,140 opioid-naïve patients who had undergone surgery in Ontario, only 168 (0.4%) continued to receive an opioid prescription one year later.^11^ Notably, the patients at highest risk of continued opioid use had undergone thoracic surgical procedures which are notorious for their high incidence of chronic postsurgical pain (i.e., these patients were most likely taking the opioids to relieve pain). Moreover, it is unclear from these data what percentage, if any, of the 0.4% who were talking opioids at the one-year mark were actually addicted.

Another recent study^12^ was so compelling that the American College of Surgeons made a public statement online in a press release^13^ stating that “opiate pain killers prescribed after severe injury do not lead to long term use,” citing a study on 7,302 patients who had sustained major traumatic injuries. Forty-nine percent filled at least one prescription for an opioid after hospital discharge and only 0.9% were still taking an opioid one year later. Although pain severity was not reported, one sequela of traumatic injury is chronic pain,^14^ so it is possible that those who were taking opioids were doing so to relieve ongoing pain. As with the above study,^11^ the authors do not report this as an addiction rate but have indicated this is the rate of individuals who were still taking an opioid one year after hospital discharge^12^ and given that the rates of persistent postsurgical pain range from 6-68%^15^ it is probable that most of these patients were using the opioid for pain and not because of an addiction.

We are not arguing that medical exposure to opioids never results in addiction but that the incidence is very low and when prescribed with appropriate precautions it is extremely rare. Fear of addiction should not be the reason for restricting opioids to the many people with chronic pain who benefit and whose quality of life is enhanced by their use. Addiction can be problematic but we have effective means of helping those individuals with chronic pain who do become dependent or addicted.^16^ In addition we are not arguing that opioids have not been overprescribed. We acknowledge that the lack of education of physicians regarding pain assessment and management and the lack of access to appropriate non-pharmacological treatments continue to contribute to cases of overprescribing. We think the solution to this is not further regulation of physician prescribing but approaches to assure improved education of health care professionals regarding pain management and improved access to care for people with pain conditions. We have previously argued that the best way to address this is through a national pain strategy.^17^

## Stopping appropriate medical prescribing of opioids will not stop people with addiction from abusing opioids and will cause significant collateral damage to people with pain

Statistics from the 2015 US National Survey on Drug Use and Health^18^ are also revealing for what they say about addiction to prescription pain relievers. The survey, comprising 68,073 face-to-face interviews, showed that 36.4% of the US population over the age of 12 years reported using prescription pain relievers and 4.7 % reported misusing them in the past year. Note, that the definition of misuse includes “use in any way not directed by a doctor, including … use in greater amounts, more often, or longer than told to take a drug …” (ref. 18, p. 9). Among the 4.7% who misused prescription pain relievers, the most common reason given for the misuse was “to relieve pain” which was reported by 62.6% of respondents with only 2.3% endorsing “because I am hooked or have to have it.” Using these numbers, it is estimated that ~0.12% of the population of the United States over the age of 12 years is addicted to prescription pain relievers.

According to the Canadian Tobacco and Alcohol and Drug Use Monitoring Survey opioid use has dropped from 20.6% of Canadians reporting use of an opioid in 2010 to 15% in 2013, while use to get high has remained at 0.2%.^19^ Curbing general use of prescription opioids will not stop people with addiction from abusing opioids. To pretend that this is so is dangerous because it leads to a false sense of security that something is being done to curb abuse. The best solution is to continue to enhance excellent care for people with addiction, a multifactorial condition requiring management of risk factors and appropriate interdisciplinary care. The Canadian Public Health Association Position Statement released in December 2016 contains an excellent set of recommendations that should be implemented.^20^

In addition, curbing appropriate medical use of opioids has already been demonstrated to harm people with pain.^21,22^ As noted above, in this anti-opioid environment many physicians are fearful of prescribing opioids at all leading to a situation where patients who benefit from an opioid are unable to get a prescription. This then leads to harm with escalating pain, decreased function and decreased quality of life. There is an increased risk of depression and suicide. In the past year, two of my (M.L.) patients have committed suicide where in the previous 25 years of my practice I am aware of only one other. Several of our colleagues have expressed similar experience and concerns. There is also a risk that some people with pain who are unable to obtain a legal prescription from their doctor will turn to the illegal market^23^ which then puts them at high risk given the dangerous highly potent synthetic opioids that are currently available.^24^ On both of these topics there are increasing patient reports and publications online supporting this collateral damage.

It is also important to recognize the burden of chronic pain relative to that of illicit substance use (e.g., cocaine, crack, amphetamines, hallucinogens, ecstasy and heroin). Chronic pain affects 20% of Canadians^25,26^ with an annual economic cost of $60 billion whereas illicit substance use affects 1.6% of Canadians^19^ with an annual cost of $8.2 billion.^27^ Also of interest is the fact that in 2013 the use of illicit substances (1.6%) was approximately half what it was in 2004 (3%),^28^ evidence that the excellent education programs against substance abuse are having an effect.

In conclusion, long-term opioids are safe and effective in the management of chronic pain when used appropriately in a significant subgroup of people. Medical use of opioids is not what causes addiction. Curbing appropriate medical use will not solve the problem of illicit opioid use or opioid related harms. In fact, the evidence supports that the current harsh regulatory climate on prescribers is doing harm to people with chronic pain. The solution is to provide enhanced timely care to those struggling with addiction and substance use disorders and better access to interdisciplinary care for people with chronic pain conditions.
